# Automatic Behavior Assessment from Uncontrolled Everyday Audio Recordings by Deep Learning

**DOI:** 10.3390/s22228617

**Published:** 2022-11-08

**Authors:** David Schindler, Sascha Spors, Burcu Demiray, Frank Krüger

**Affiliations:** 1 Institute of Communications Engineering, University of Rostock, 18119 Rostock, Germany; 2 Department of Psychology, University of Zurich, 8050 Zurich, Switzerland; 3 University Research Priority Program “Dynamics of Healthy Aging”, University of Zurich, 8050 Zurich, Switzerland; 4 Faculty of Engineering, Wismar University of Applied Sciences, 23966 Wismar, Germany

**Keywords:** social behavior analysis, uncontrolled audio recording, deep learning, wearable device

## Abstract

The manual categorization of behavior from sensory observation data to facilitate further analyses is a very expensive process. To overcome the inherent subjectivity of this process, typically, multiple domain experts are involved, resulting in increased efforts for the labeling. In this work, we investigate whether social behavior and environments can automatically be coded based on uncontrolled everyday audio recordings by applying deep learning. Recordings of daily living were obtained from healthy young and older adults at randomly selected times during the day by using a wearable device, resulting in a dataset of uncontrolled everyday audio recordings. For classification, a transfer learning approach based on a publicly available pretrained neural network and subsequent fine-tuning was implemented. The results suggest that certain aspects of social behavior and environments can be automatically classified. The ambient noise of uncontrolled audio recordings, however, poses a hard challenge for automatic behavior assessment, in particular, when coupled with data sparsity.

## 1. Introduction

Being able to live an active and meaningful social life well into old age is a significant predictor of psychological well-being and longevity [1,2]. Having few social relationships and infrequent social interactions has been associated with unfavorable health outcomes [3]. The quantity and quality of people’s daily interactions with others have a significant impact on their well-being [4]. In order to accurately gain information on human social behavior and its relation to well-being, it is necessary to measure individuals’ overall social activity participation, and how they engage in social activities in everyday life across time and contexts. It is not optimal to use the traditional survey method or self-report-based ambulatory assessments due to their limitations, such as memory bias, response styles, demand characteristics, social desirability, and limitations to introspection [5]. A method that operates with unobtrusive observation of individuals and the objective coding of their real-life behavior would be desirable for social behavior analysis. For observation, audio data could be recorded during the daily life of the participants as, for instance, done by the EAR system [6]. Coding of the behavior is typically done in a manual process by multiple domain experts to prevent subjective ratings, resulting in an expensive process. By supporting this process with automatic behavior classification from audio recordings, these costs could be reduced while maintaining a certain degree of objectivity. Moreover, by employing light-weight classification methods, parts of the automatic coding could directly be done on the recording device, eventually enabling real-time analysis and intervention while satisfying privacy constraints at the same time.

In this article, a classification pipeline, based on pretrained representations and multi-head attention is proposed to address the problem of social behavior classification from uncontrolled audio recordings. In contrast to controlled audio recordings, where different factors, such as background noise and distance to the microphone, are fixed and known, uncontrolled audio recordings provide differences in all aspects, posing a hard challenge for automatic audio classification. The proposed approach is evaluated on a dataset consisting of 30 s audio snippets randomly recorded during the daily life of 93 participants. Typical problems contained in the dataset are, for instance, that the recording device was not in close proximity or the voice was covered by friction or other background noise. Moreover, the dataset is imbalanced by orders of magnitude. The results suggest that information about social behavior can indeed be inferred from uncontrolled audio recordings. The *Activity*, for instance, could be recognized with an F-Score of 0.68.

The contribution of this article is two-fold:

1. The problem of classifying social behavior and environment from uncontrolled audio recordings is introduced. The major challenge in the explored setting results from the mode of recording and annotation which is further explored in Section 3.1.
2. An initial solution to the problem is proposed by use of pretrained audio representations in combination with an attention mechanism.

The article is structured as follows. Section 2 gives an overview of related work. Section 3 outlines the used dataset, provides further details on the given problem, and describes the proposed classification pipeline, including methods for optimization and evaluation. Evaluation results are presented in Section 4 and discussed in Section 5. Finally, Section 6 concludes and provides an overview of future work.

## 2. Related Work

Yordanova et al. [7] investigated how social behavior and environment coding can be performed based on human generated, textual transcriptions of audio recordings based on the same dataset as used in this work (see Section 3.1). A bag-of-words model showed promising results for automatic coding when combined with data augmentation. However, the approach has the drawback of being dependent on a given transcription including a speaker identification. Due to the uncontrolled recording, speech recognition is difficult (see Section 3.1.1) and a manual transcription of the audio files is not feasible for intended applications. Moreover, speech is not present in a majority of samples with only ≈20% of recordings having a transcription (see Section 3.1.1). This work explores automatic coding based directly on uncontrolled audio recordings.

### 2.1. Audio Analysis

With respect to the automatic classification of audio samples, Convolutional Neural Networks (CNNs) are often employed. They are adapted from computer vision [8], where CNNs consistently outperform other methods on benchmarks such as ImageNet [9]. CNNs for audio classification are typically applied to spectral representations of audio signals [8,10,11], such as the Mel-spectrogram based on the Mel-Scale [12], created to capture human audio perception. Different tasks relate to the automatic analysis of audio recordings and to the task at hand.

Audio Event Detection (AED) addresses the identification of particular events within audio sequences [13,14,15]. It is related to the problem at hand as all considered classes (see Section 3.1) are characterized by audio events. AudioSet [16] is the largest available dataset for AED with 2 M samples of 30 s, collected from Youtube, but has the drawback of requiring data to be downloaded from Youtube which leads to samples gradually disappearing as well as usage right issues [17]. The *AudioSet Ontology* covers 632 different sound classes and structures them hierarchically. Spectrogram-based CNNs currently achieve state-of-the-art performance on AudioSet AED [11]. Most recently, FSD50K [17] was introduced as an open data corpus based on 200 classes from the *AudioSet Ontology* to overcome the usability issues of AudioSet. It contains 51 k samples with sample lengths ranging from 0.3–30 s.

Speech Emotion Recognition (SER) is concerned with classifying the emotion conveyed by a speaker. It is related to the given task in certain aspects, for instance, for inferring the mood or the conversation type of a person from their voice (see Section 3.1.1). SER is typically applied in controlled laboratory settings with trained speakers and pre-selected utterances [18,19]. The dataset at hand, however, consists of uncontrolled audio recordings where only ≈20% of the samples contain speech. Thus, approaches for SER cannot directly be applied.

Automatic Speech Recognition (ASR) deals with the automatic decoding of speakers’ utterances into text. In the given setting, ASR could be beneficial to the task by allowing a semantic interpretation of speech, e.g., for determining the conversation type mentioned above (see also Section 3.1.1). However, the spoken language in this context is Swiss German, a low-resource language for ASR [20], for which pretrained models are not available. Therefore, ASR is not considered in this work.

### 2.2. Transfer and Representation Learning

The idea of representation and transfer learning is that training data and test (or application) data do not have to be identically distributed [21], instead general representations are learned on large databases and then transferred to related problems with few data available. For computer vision, a common approach is to pretrain models on ImageNet and fine-tune them to other computer vision tasks. In Natural Language Processing (NLP), language models such as BERT [22] are pretrained with self-supervision on large corpora in order to learn a suitable representation to be applied for multiple different downstream tasks.

The idea of representation and transfer learning for audio has recently gained interest in the scientific community. *Wav2Vec*, for instance, is an embedding method where self-supervision is used for training a CNN on raw audio input with the objective of distinguishing the true future audio from false samples [23]. The embedding features generated by Wav2Vec can replace spectral representations as input for downstream tasks and have been reported to improve performance [23]. It was further improved in Wav2Vec 2.0 [24] by replacing the convolutional architecture by one initial temporal convolution followed by a transformer [25]. Moreover, the objective is changed from predicting a future sequence to partially masking a given sequence and distinguishing the masked snippet from distractors. Liu et al. [26] proposed learning speech representations with bidirectional transformer encoders based on predicting masked frames in mel-spectrograms based on left and right context. The work is extended by Chi et al. [27] who adapted ALBERT—a transformer-based model known from the NLP domain—that reduces the number of required parameters. While the methods described above are potentially applicable to audio data in general, they are speech-specific and were only evaluated on ASR, e.g., Wall Street Journal [28], LibriSpeech [29].

Other work has focused on generating general audio representations not primarily focused on speech. Pretrained Audio Neural Networks (*PANNs*) adapt and develop CNN architectures for AED on AudioSet and achieve competitive performance [11]. They map 10 s audio samples down to an embedding vector that can be used for transfer learning. Most of the proposed PANNs use only Mel-spectrogram representations of a given input while one proposed architecture learns features directly from the given audio sequence as well as the Mel-spectrogram. Since the pretraining is performed on labeled AudioSet data, it is performed in a supervised manner. Recently, there have been further studies investigating the generation of general audio representations with unlabeled data by utilizing self-supervision. Saeed et al. [30] used contrastive learning by assigning high similarity to audio segments extracted from the same recording while assigning lower similarity to segments from different recordings. Niizumi et al. [31], in contrast, did not use negative samples, but instead employed a method named Bootstrap Your Own Latent, which directly minimizes the mean squared error of embeddings originating from the same input with contrasts created by data augmentation. Further, Wang et al. [32] proposed an evaluation suite for Audio Representations covering 12 tasks set in the domains of general audio, speech, and music.

### 2.3. Mobile Networks

Mobile networks aim at reducing the size and execution complexity of neural networks in order to make them applicable in real world settings where computational resources are limited or a timely computation is required [33]. For the task at hand, this is relevant since the target application could be integrated with the data recording directly on participants’ mobile devices. Two different network architectures with reduced complexity have been adapted to audio classification as PANNs [11]:

1. *MobileNet* that uses depth-wise separable convolutions to reduce complexity [33], and
2. *MobileNetV2* that extends MobileNet by an inverted residual structure with shortcut connections [34].

## 3. Materials and Methods

### 3.1. Dataset

The dataset was recorded with an Electronically Activated Recorder (EAR) [6] and captures 30-s audio recordings collected during the daily life of participants. The EAR is implemented as a smartphone app and is thus able to unobtrusively track real-world behavior by periodically recording ambient sounds [35] from the respective devices’ standard microphones. Audio data were recorded for two groups of healthy participants:

1. Young adults (n = 61, 23 m, 38 f, average age 25), and
2. Older adults (n = 32, 12 m, 20 f, average age 72).

The dataset is intended for comparative psychology studies and, therefore, comprises both age groups. Social behavior for these groups is, in general, different, but only within the limits of the classes of interest, as described below. For instance, “watching TV” is expected to be similar between both groups, while more variance is anticipated in “walking”. We argue that this variance is essential for generalization of our approach as it leads to a broader range of data, preventing the classifier from overfitting.

Participants of both groups live in Switzerland and their mother tongue is Swiss German. All participants wore the EAR system for a period of 4 days for which it randomly recorded 286 samples per participant on average. During the recording period participants were asked to keep a diary that was later used to support annotation. The original data collection resulted in 26,885 audio files, 18,039 for the young adults and 8846 for the older adults. Some participants later withdrew their consent reducing the final number of available samples to 22,439 (14,398 and 8041) corresponding to 187 h of recordings.

For performance evaluation, the dataset was divided into three sets:

1. *Training*;
2. *Development*;
3. *Test*.

Both sets, young as well as older adults, were split separately in approximately a 3:1:1 ratio resulting in a combined count of 14,124:4501:3814 samples for *train*, *development*, and *test* set. The sets were split based on persons by assigning all available samples for a single person to one of the datasets in order to guarantee generalization in the evaluation.

#### 3.1.1. Annotation

Recorded audio samples were coded by domain experts with a background in psychology, after receiving detailed annotation instructions and training on this task. Recordings were coded with respect to multiple categories capturing social behavior and the participants’ environment. The categories as well as the respective codes were identified by the 3rd author who is an expert psychologist researching healthy aging [36]. Each recording was handled by two annotators responsible for different coding categories. For both participant groups, the inter-rater reliability was above 80%, indicating almost perfect agreement.

**Figure 1:**
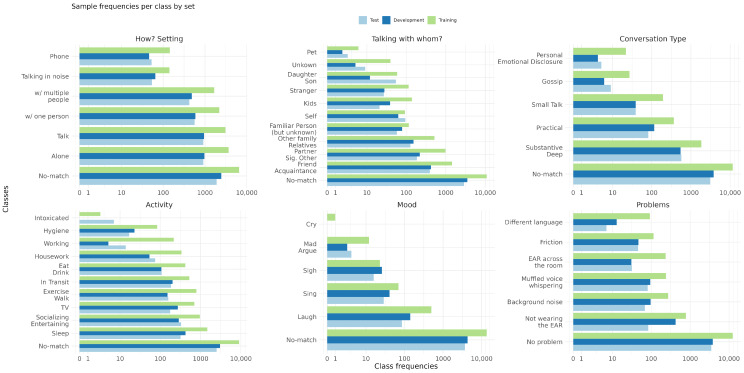
Overview of the sample sizes per surrounding-based coding category split into *train*, *development*, and *test* set. Additionally to the categories, problems existing in samples are also coded. Sizes are depicted on a logarithmic scale.

The coding categories considered for automatic coding including their classes are summarized in Figure 1:

- *How? Setting* captures the social setting by determining whether other people are present;
- *Talking with whom?* identifies the current conversation partner of the participant;
- *Conversation Type* identifies the type of conversation between the participant and the conversation partner;
- *Activity* describes the participant’s current activity;
- *Mood* captures the current mood of the participant.

Moreover, *Problem* is included as an additional category, even so it does not correspond to a psychological category, because knowledge about annotation problems can add value to the automatic coding by identifying samples with insufficient audio quality.

In this work, we focus on categories that can be classified based on ambient sounds or voice-based features such as pitch, intonation, talking speed, and length of speech intervals. We exclude further annotated categories that would require the semantic interpretation of spoken words. While the category *Conversation Type* was also annotated based on semantic interpretation of words, it is included because we argue that some distinctions between the given classes could be made based on voice features only, for instance, distinguishing small talk from a deep conversation.

##### Classes

**Figure 2:**
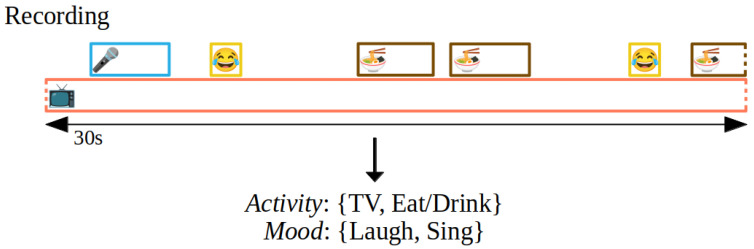
Illustration of a potential sample. There is a continuously present background noise in form of a TV, a short singing period, two isolated laughs as well as eating. The goal is to correctly assign the *Activity* labels *TV* and *Eat/Drink* as well as the *Mood* labels *Laugh* and *Sing*. Further, all other labels have to be correctly identified as negative. A potential danger is to mistake the TV for a second person in the room assigning a wrong *Setting*.

The classes corresponding to all coding categories are not mutually exclusive. For each class, assigning no code to a sample is a valid option, and it is also important to automatically recognize those cases. Therefore, an additional *No-match* class is added for each category as illustrated in Figure 1. The coding scheme has no claim to be exhaustive for any of the categories and annotators were instructed to only annotate classes when they are certain. For instance, annotators were instructed to make no annotation if they hear a radio or cannot distinguish between the given class *Activity:TV* and a radio. Further, small indicators are sufficient for class annotation, e.g., for the class *Mood:Laugh*, the annotation instruction states: *“This can be one short laugh”*. Due to the long duration of a single sample, this makes coding a complex problem that is closer illustrated in Figure 2.

##### Background Knowledge

Annotation was supported by a diary that participants have kept throughout their recording period. It is particularly important for the category *Talking to whom?* and classes such as *Activity:Sleep*. Annotators were instructed to prioritize sound over diary if they find contradicting indications. Further, annotators were aware of participants’ demographics and their living situation, and encouraged to make use of this information for annotation. To a certain degree, temporal dependencies between recordings are also considered, e.g., given that a participant is watching TV in a recording at 8.30 p.m. and at 8.35 p.m., an annotator will assume an ongoing activity at 8.33 p.m. if no different indication is found in the recording.

##### Issues from Uncontrolled Audio Recordings

Issues in understanding are explicitly coded as illustrated in Figure 1. If problems are present, annotators are encouraged to only code classes they can code with absolute certainty regardless of the indicated problem. Typical issues include muffled voices, friction or dampened sound in general. Those issues exist because participants do not continuously carry their phones, or have them placed in purses, pockets or backpacks. Another common occurrence is background noise, e.g., caused if participants are out in public. In total, audio problems were annotated 2764 times in the given 22,439 samples (≈12%). Samples that were not explicitly annotated to contain an acoustic problem can, however, still contain a less intense version of such problems. All described problems directly hamper classification as they influence audio quality.

Annotators also performed a speech transcription as part of the annotation. Quantifying problems they encountered performing the speech transcription gives further insights into audio data quality. In total, only 4490 (≈20%) samples have a transcription; however, background chatter can still be present in the remaining samples as only conversations of the participant are transcribed due to ethical restrictions. For the remaining samples, classification can only be based on ambient sounds. Utterances within speech transcriptions cannot always be clearly understood. Within a total of 2141 (≈48%) transcriptions, a problem is indicated. At least one word was not understood in 1581 (≈35%) transcriptions while an entire sub-sentence was not understood in 909 (≈20%). Overall, 3140 words and 1360 sub-sentences could not be transcribed.

### 3.2. Problem Statement

The objective of this work is to investigate whether an automatic coding as outlined in Section 3.1.1 can be established. The class *Activity* serves as main classification target, as it mainly reflects the participants’ behavior. Further, the resulting application is intended as a real-time application on mobile devices with limited computational resources. Therefore, a main aspect of this work is to investigate how its complexity can be reduced and how strongly performance will be influenced.

Input is an audio sample x of 30 s length represented in a waveform. For each category $C_{i}\in C_{1},\ldots ,C_{N}$, all classes $c_{j}\in c_{1},\ldots ,c_{len(C_{i})}$ are classified between *no-match* (=0) and *match* (=1). The classification of each category is considered as a separate multi-label problem because the coding is not mutually exclusive. Codes are assigned to the entire 30 s audio snippet x because for the target application, it is not relevant to find the precise onset of audio events but to indicate their existence. In addition to the original codes, the category *Problems* was introduced to reflect the different problem types.

### 3.3. Automatic Coding

The objective of this work is the automatic coding of audio recordings based on the categories described in Section 3.1.1. As described, labels are assigned to entire sequences of 30 s and all problems are multi-label classifications.

#### 3.3.1. Preprocessing

Audio samples are preprocessed to conform with the pretraining procedure of PANNs used for feature extraction. In a first step, all audio samples are resampled from 44 kHz to 32 kHz. This step reduces the range of covered frequencies but still covers the average range of human hearing. Then, audio samples are transformed into logmel spectrograms with a window size of 1024 samples (32 ms), a hop length of 320 samples (10 ms), and 64 mel-bins are used, which has been shown as a good trade-off between complexity and performance [11].

#### 3.3.2. Data Augmentation

The number of available data samples is massively imbalanced between the classes of the individual categories, see Figure 1. For the underrepresented classes, there are often only few samples available and they are, therefore, hard to learn and distinguish for a classifier. Data augmentation is a method to artificially increase the number of available samples by automatically generating new samples from given ones [8]. Different methods for data augmentation of both, audio samples and spectral representations are applied here:

- *Pitch shifting* raises or lowers the pitch of samples while keeping length unchanged [8].
- *Time stretching* slows down or speeds up a sample while keeping the pitch unchanged [8].
- *Noise injection* adds white noise. Salamon et al. [8] proposed adding background noises such as urban sounds. However, for the given problem, background sounds are important features for the classification.
- *Spectrogram masking* randomly masks continuous spans in time and frequency domains of generated logmel spectrograms and is included in the training of PANNs [11,37].

The first three are collectively applied on the raw audio signal, while the latter is employed individually on the spectral representation. In this work, raw audio augmentation is implemented dynamically with random initialization based on Gaussian distributions in order to vary the strength of the augmentation on sample basis during the entire training process.

#### 3.3.3. Classification Pipeline

The pipeline for audio classification consists of:

1. Splitting into overlapping sub-sequences;
2. Feature extraction for each sub-sequence;
3. Merging of feature representations;
4. Classification based on combined feature representation.

**Figure 3:**
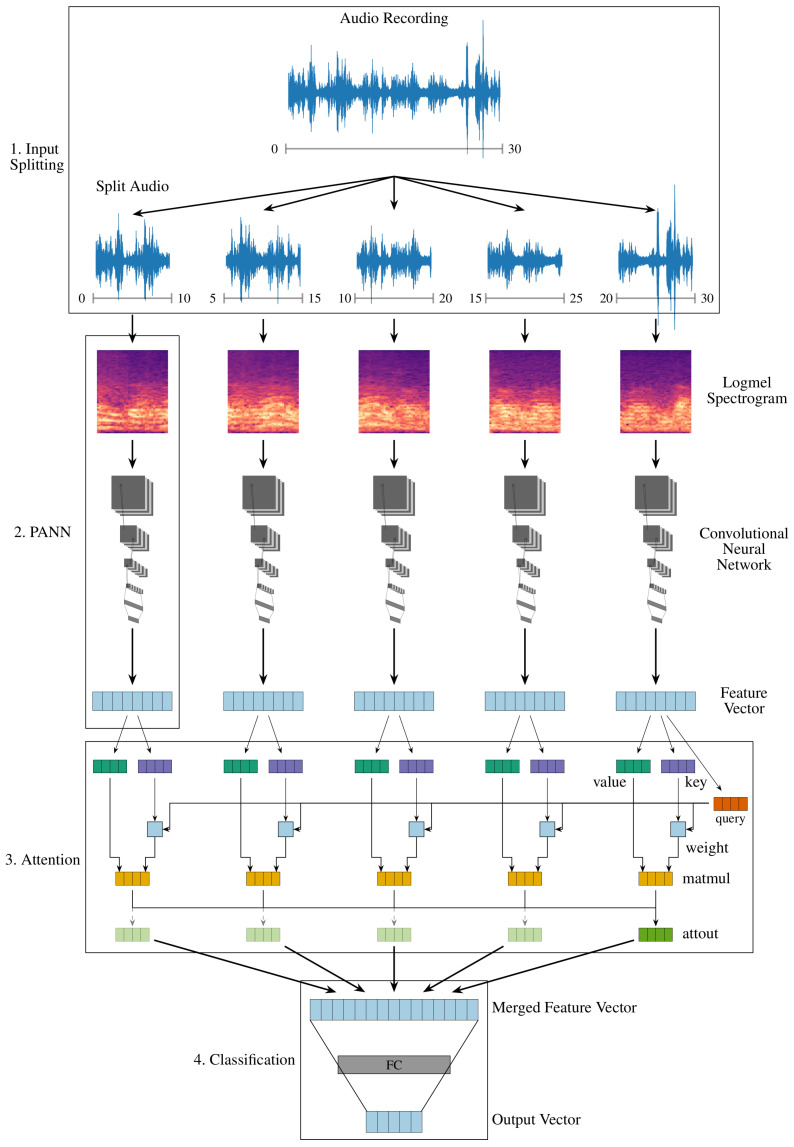
Classification example for a single 30 s audio sample. Initially, it is split in overlapping 10 s segments. Next, a logmel-spectrogram is generated from the sample which is processed by a PANN (see Section 2). Merging of PANN feature representations is illustrated by an attention mechanism. Attention outputs are averaged and the final feature representation is classified by fully connected layers. The attention mechanism is illustrated for the first feature representation, but is calculated the same way for each feature.

It is illustrated in Figure 3.

##### Sequence Splitting

The dimension of the spectral representation of the audio samples is given by the number of mel-bins (e.g., 64, see Section 3.3.1), but—assuming a fixed window size—the time domain depends on the length of the input sequence. As described in Section 2, the available pretrained PANNs employ audio sequences of 10 s length. To this end, audio sequences are split into a length of 10 s, to achieve consistency with AudioSet (see Section 2). A 50% overlap of 5 s is used for splitting to make sure all audio events are represented and not accidentally split. The process results in 5 × 10 s for each 30 s recording and is illustrated in Figure 3 (1. Input Splitting).

##### Sequence Feature Extraction

PANNs [11] are used to extract features from the split audio sequences, see Figure 3 (2. PANN). The networks contain valuable feature representations for the given task because they were pretrained on AudioSet and achieve state-of-the-art performance [16]. AudioSet is currently the largest supervised pretraining basis for audio events with over 2M samples. Moreover, many AudioSet ontology classes are related to the given coding classes (see Section 2). For instance, the class *Mood:Laugh* can be well related to the AudioSet class *Laughter*, represented as: *Human Sound* → *Human voice* → *Laughter* with subcategories: *Baby laughter*, *Giggle*, *Snicker*, *Belly laugh*, and *Chuckle, chortle*. Other categories and classes such as *Conversation Type*: *Deep Talk* cannot directly be related. PANNs are selected over other general audio representations described in Section 2 because there is a range of different models available that allow to test the effect of parameter reduction for mobile applications. However, it is necessary to further explore the influence of the quality of audio representation on the classification in future work.

PANNs are convolutional neural networks and are available pretrained with different layer architectures and complexities. Some are adapted from well performing computer vision architectures while others were newly established. The ones considered here are summarized in Table 1.

They were chosen based on two aspects:

1. Pretraining performance on AudioSet;
2. Model complexity with respect to the number of parameters.

The main focus was set to PANN MobileNets, as they are optimized to work with a low number of parameters and to reduce execution complexity [11,33,34], which makes them particularly interesting due to their mobile application capabilities. Other PANN configurations were considered to estimate the influence of the reduced number of parameters.

Since the weights in PANNs are pretrained and do, therefore, contain valuable representations for input samples, the influence of those weights is investigated. To this end, the following three options were considered:

1. **Freeze** pretrained weights and perform no updates to the PANNs based on the EAR data;
2. **Fine-tune** weights based on new data;
3. **Gradually unfreeze** weights from the top to the bottom layer in order to first adjust most complex operations and simple filters last, as proposed by Howard and Ruder [38].

##### Feature Merging

Individual feature representation for the 10 s splits have to be combined to classify the entire audio sequence of 30 s, see Figure 3 (3. Attention). Three different strategies for merging the features are investigated here:

1. **Concatenation** of feature representations;
2. **Averaging** of feature representations;
3. **Weighting** feature combinations **by attention**.

While concatenation and averaging are basic mechanisms to combine features, an attention mechanism has the ability to assign higher weights to relevant parts of the sequence that contain the best acoustic clues for certain classes. The used attention mechanism is based on the attention principle proposed by Bahdanau et al. [39] and implemented with multiple attention heads as in Vaswani et al. [25]. The mechanism for a single attention head is illustrated in Figure 3, and the number of used attention heads is optimized. Eight attention heads are used as default as reported for the Transformer [25]. The number is not further increased because each attention head greatly increases the computational complexity and a value of eight already results in ≈1.6 M trainable model parameters.

##### Classification

Additionally to the PANN-generated features, information on time and weekday of the recording is added for classification. They are encoded in a standard approach for representing circular features according to Equation (1) in order to ensure correct closeness between times such as 11 p.m. and 1 a.m.

(1) $$v_{1}=\sin\left(\frac{\pi t}{t_{max}}\right),v_{2}=\cos\left(\frac{\pi t}{t_{max}}\right)$$

The information on time and day is also available in the target application and is assumed to have a positive effect on specific classes where time influences the prior probability, e.g., *Sleep*.

Finally, classification on merged features is performed by applying fully connected layers which map the feature representations down to the number of output classes per category, see Figure 3 (4. Classification). A sigmoid activation function is used to map the output for each class to a range between 0–1. Therefore, a threshold for classifying samples needs to be determined that influences the trade-off between recall and precision. For evaluation during optimization, the threshold is set to a fixed value of 0.5. To evaluate the final performance, the threshold is determined individually for each class based on the optimal setting from the development set.

##### Multi-Task Learning

The given automatic coding problem covers multiple categories (see Figure 1), which can either be handled individually by training a separate classifier for each task or handled in parallel as a multi-task learning problem by sharing layers and weights between tasks. A multi-task model has the advantage of reducing the overall execution cost because shared layers only have to be calculated once for all tasks. Moreover, multi-task learning can improve recognition performance and help learn better representations if the given tasks are related, as it implicitly increases the sample size [40]. Therefore, the representations learned by a model might generalize better because the influence of task-specific noise is reduced. If there is no relation between problems, the overall number of parameters has to be split between unrelated tasks and performance is likely to decay. We assume that the given tasks are related as, for instance, information from Activity recognition can be useful for each other task. Moreover, all tasks are—to varying extends—related to human speech.

The proposed model architecture shares all weights for the feature extraction which corresponds to sharing the entire employed PANN. Since the features trained in the PANNs are successful in classifying according to the broad AudioSet ontology, it is assumed that the different classes of the given problem can also be represented through shared weights. A separate attention layer for each task is employed for merging because acoustic events for different categories can be contained in different sub-sequences. The other merge methods described under *Feature Merging* are static and equal for all categories, therefore, they are inherently shared. The remaining fully connected layers for classification are category-specific and do not share weights.

The weights in a neural network are updated based on a loss function evaluating the network output against a target output. For the given multi-label categories, *Binary Cross Entropy* loss is applied. In multi-task learning, every individual task has its own loss function, and the combination of individual losses needs to be considered for optimization. Plain addition of weights might bias the optimization by giving high weights to classes for which the loss is high due to structural problems, e.g., classes cannot be distinguished because a used method is not suited to represent the problem. Weighting the losses can counteract this influence, but it is challenging to manually assign meaningful weights. Loss weights for different tasks are learned during training as proposed by Kendall et al. [41].

##### Training and Optimization

To train the classification model, Adam [42] and Adagrad [43] optimizers are tested. Adam was used to pretrain PANNs, and both optimizers offer advantages for training imbalanced problems with small classes [44]. Dropouts [45] and Batch Normalization [46] have been shown to work well for regularization in neural networks. Both mechanisms are included in training PANNs and also used in fine-tuning. PANN dropouts are optimized and an additional dropout layer is applied on the generated PANN feature representation. Early-stopping is performed to prevent the model from overfitting when both macro binary F-Score and weighted binary F-Score on the development set did not improve for at least 9 consecutive epochs. The value was chosen empirically. In case macro and weighted average were found to be contrary, training is ended when their average did not improve or maximum epochs are reached. As *Activity* was selected as main classification target (see Section 3.2), it was also selected as optimization target for multi-task learning.

All implementations were performed in PyTorch [47] 1.5.1 and the code is made publicly available upon acceptance.

#### 3.3.4. Evaluation

Four evaluation, scores are considered for the multi-label classification categories.

**Correctly classified samples**: samples are counted as correctly classified for one category if all class labels are assigned correctly. This allows a basic performance estimation, but is susceptible to class imbalances.

**Hamming score** [48,49]: calculates the binary distance between given target labels *T* and assigned predictions *P* for a given sample. It can be considered as a score for multi-label accuracy. It is susceptible to class imbalance.

$$H=\frac{1}{n}\sum_{i=1}^{n}\frac{\left|T_{i}\cap P_{i}\right|}{\left|T_{i}\cup P_{i}\right|}$$

**Multi-label precision, recall** [48,49]: calculates precision and recall between the target labels of a sample *T* and its predicted labels *P*. The score is average across all samples. This score is also susceptible to imbalance.

$$p=\frac{1}{n}\sum_{i=1}^{n}\frac{\left|T_{i}\cap P_{i}\right|}{\left|T_{i}\right|},r=\frac{1}{n}\sum_{i=1}^{n}\frac{\left|T_{i}\cap P_{i}\right|}{\left|P_{i}\right|}$$

**Class-based binary evaluation**: every class in a sigmoid multi-label problem can be analyzed as a binary problem for which standard metrics can be calculated. Those metrics allow to assess how well each individual class can be recognized. For each class, *precision*, *recall*, *f-score*, *sensitivity*, *receiver operating characteristics (ROC)*, and *precision-recall curve* are provided. Individual class scores are summarized by macro and weighted average for each category to evaluate the performance for an entire category. Through this score, the influence of class imbalance can be evaluated.

## 4. Results

In the following, the classification results are reported and influences of different hyper-parameters compared. The default parameter configuration—referred to as base model—is summarized in Table 2.

### 4.1. Optimization

In Section 3.3, different network architectures and optimization options for the given task were described. To make a sound decision for the best design option, every aspect was systematically evaluated based on the development set. Due to the high time complexity, every design option was evaluated individually rather than performing an exhaustive grid search. *Activity* was chosen as a representative task and optimized with a selected base configuration. The configurations and results are summarized in Table 3. Note that the F-Score for *Intoxicated* was counted as zero in the binary macro average because no samples are present in the development set.

#### 4.1.1. Pretraining

The results showed that, overall, usage of pretrained models has a positive effect on recognition performance (see Table 3 *Base Model: pretrained* and *untrained*). The number of overall correct classifications notably improved. Moreover, there is also an improvement in the weighted binary F-Score. It was further found that pretraining leads to a notable decrease in required training time, but also leads to earlier and stronger overfitting.

#### 4.1.2. Optimizer

In general, both Adam and Adagrad showed similar results with the exception of binary macro F-score where Adam performed better (see Table 3, *Base Model* and *Optimizer*). Moreover, Adam was found to reduce overfitting in combination with pretrained models as compared to Adagrad, and is, therefore, selected as the preferred choice. For multi-task learning, Adam was found to perform better across all scores compared to Adagrad (see Table 3, *Multi-task* and *Base Model*).

#### 4.1.3. Dropout

A higher dropout value was found to reduce the overall number of correct classifications while recognition of classes with a low number of samples (see Table 3, *Base Model* and *Dropout*) improved, which led to a better binary macro score. Moreover, higher dropout values were observed to slightly reduce overfitting. In the selected model setup, dropouts are not applied because of the decrease in overall classification performance.

#### 4.1.4. Freezing

Freezing the pretrained weights of PANNs requires less training time but also leads to faster overfitting. Additionally, freezing has a negative influence on category scores compared to fine-tuning (see Table 3, *Weight Freezing* and *Base Model*). Gradual unfreezing was found to notably reduce all scores.

#### 4.1.5. Feature Merging

Concatenation of PANN feature representations was found to reduce performance across all scores while averaging feature representations leads to a reduction in category scores. Therefore, attention is chosen as the preferable way to combine feature representations (see Table 3, *Feature Merging* and *Base Model*).

#### 4.1.6. Attention Heads

With a lower number of attention head, and a corresponding reduction in model parameters, overfitting is reduced. However, fewer attention heads also lead to an overall decrease in performance (see Table 3, *Attention Heads* and *Base Model*). Therefore, 4 attention heads were chosen as a trade-off.

#### 4.1.7. PANNs

Application of PANN *Cnn10* was found to reduce category scores while binary F-Scores were almost identical, even so the *Cnn10* architecture has more trainable parameters than the *MobileNetV2* architecture. Through the *Wavegram-Logmel-Cnn14* architecture, both binary scores were improved while category scores showed a performance decrease. A larger parameter space for feature generation was not found to improve overall results, making *MobileNetV2* the preferable choice (see Table 3, *PANN Model* and *Base Model*).

#### 4.1.8. Data Augmentation

Audio augmentation was found to benefit performance across all tasks and to reduce overfitting. Spectrogram augmentation was found to improve the binary macro F-Score but, at the same time, to reduce all category scores (see Table 3, *Augmentation* and *Base Model*). Therefore, data augmentation was performed directly on audio samples.

#### 4.1.9. Multi-Task Learning

The application of multi-task learning in combination with the Adagrad optimizer was found to decrease performance across all scores (see Table 3, *Multi-task* and *Base Model*). For multi-task learning with Adam, increased performance for binary scores was found (see Table 3, *Multi-task* and *Optimizer*). These findings support the choice of the Adam optimizer and show that multi-task learning can be applied without decreasing performance.

### 4.2. Final Classification Model

Based on the optimization findings, the base classification pipeline was adapted as follows:

- Adam is used as optimizer instead of Adagrad;
- Four attention heads were chosen instead of eight;
- Audio data augmentation is performed.

As positive performance effects are not necessarily additive, the selected architecture was also tested on the development set. It was found that for multi-task learning, audio augmentation decreases performance compared to plain optimization by Adam (see Table 3, *Multi-task* and *Selected*). A possible reason why the positive effect of audio augmentation is mitigated could be the implicit increases of sample size resulting from multi-task learning. Therefore, audio data augmentation is not performed and the final model is defined as the base model adapted to multi-task learning, employing the Adam optimizer for training.

### 4.3. Performance Estimation

**Figure 4:**
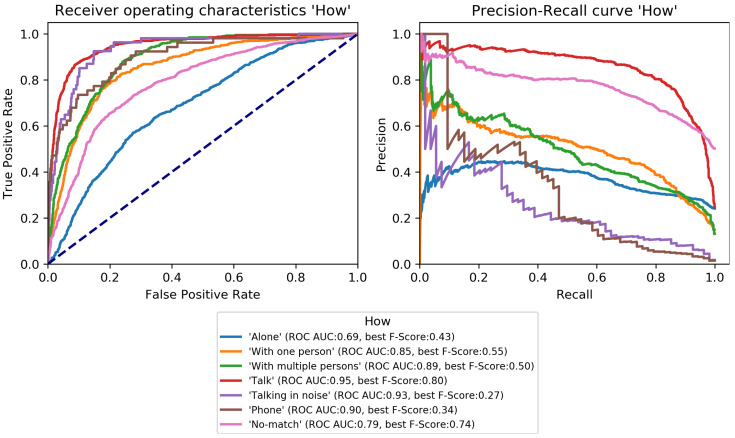
Receiver-operating characteristics and precision-recall curves for all binary classification targets of *How? Setting* (see Figure 1).

**Figure 5:**
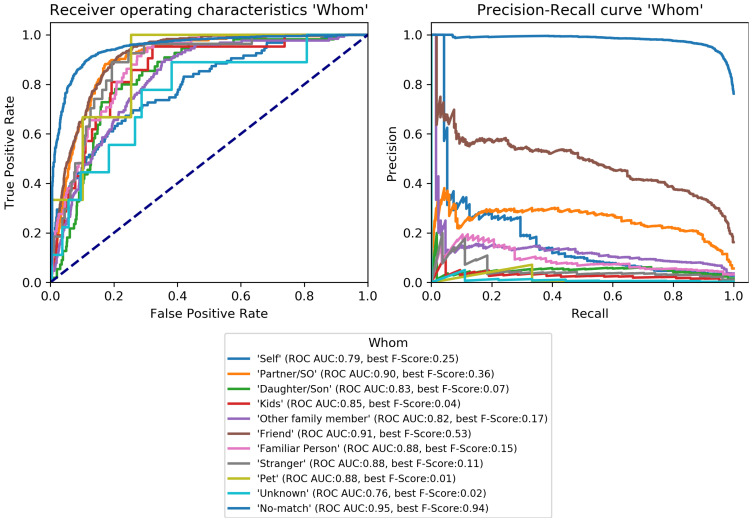
Receiver-operating characteristics and precision-recall curves for all binary classification targets of *Talking with whom?* (see Figure 1).

**Figure 6:**
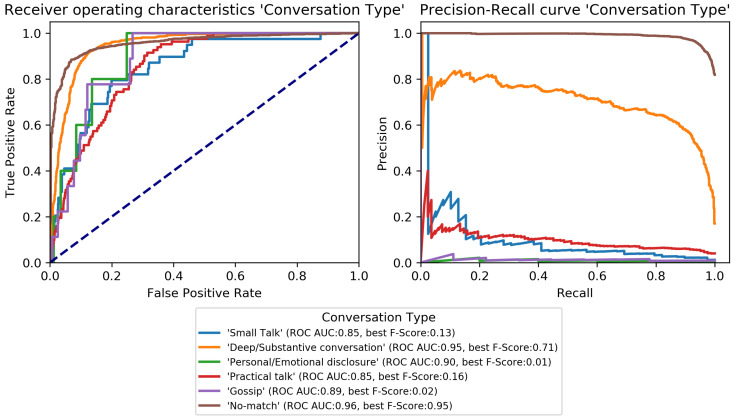
Receiver-operating characteristics and precision-recall curves for all binary classification targets of *Conversation Type* (see Figure 1).

**Figure 7:**
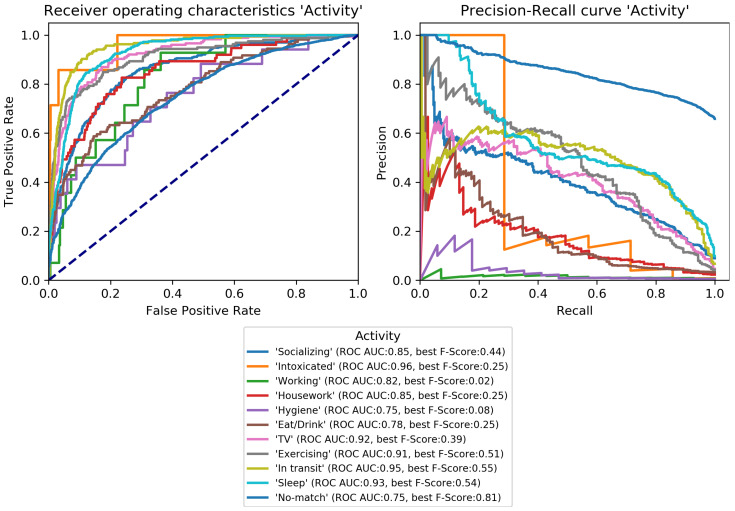
Receiver-operating characteristics and precision-recall curves for all binary classification targets of *Activity* (see Figure 1).

**Figure 8:**
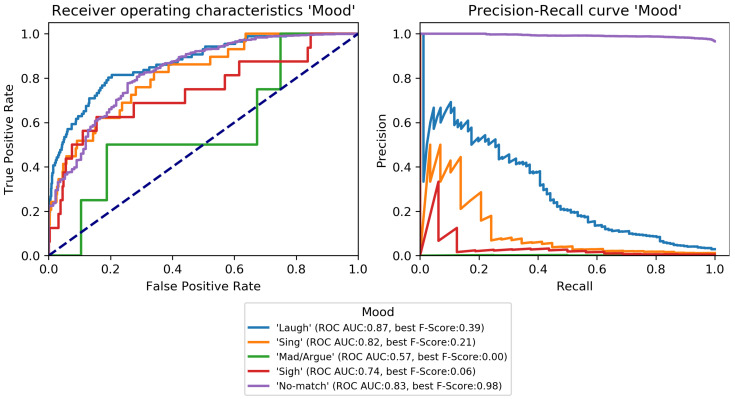
Receiver-operating characteristics and precision-recall curves for all binary classification targets of *Mood* (see Figure 1).

**Figure 9:**
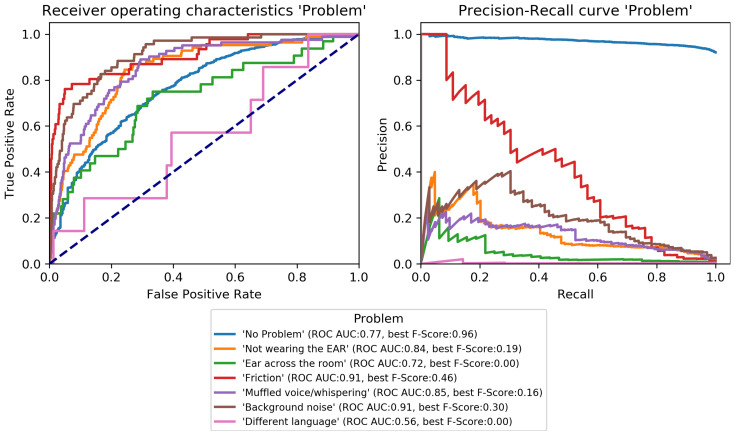
Receiver-operating characteristics and precision-recall curves for all binary classification targets of *Problem* (see Figure 1).

The final classification model was trained on the train set and evaluated on the development set to perform early-stopping and to select class-specific thresholds (see Section 3.3.3). Early-stopping was applied after 31 training epochs. The model architecture was then trained again on the combined train and development set for 31 epochs and its performance estimated on the test set. Performance scores per category are given in Table 4 and class-specific binary results in Figure 4, Figure 5, Figure 6, Figure 7, Figure 8 and Figure 9. In difference to the scores given in Table 3, all scores are calculated based on optimized thresholds. In comparison, this leads to a notable decrease in correct samples for *Activity* and a notable increase in hamming score as well as sample-based F-Score which is due to most thresholds being shifted towards higher recall. In summary, the threshold adjustment has a positive influence, as it leads to a higher number of correctly classified labels. Performance on the development set is, in general, better than for the test set with the exception of the category *Problem*.

The percentage of correctly classified samples, the hamming score and the sample-based F-Score given in Table 4 show that a large number of samples and labels per category are classified correctly. However, most category scores—except the binary macro average—are biased by class imbalances. An overview of the class-specific performance can best be gained from ROC and precision-recall curves given in Figure 4, Figure 5, Figure 6, Figure 7, Figure 8 and Figure 9. Here, it was found that performance varies notably between categories. For *Activity* and “*How? Setting*”, multiple classes can be recognized with a good recognition performance while for *Conversation Type* and “*Talking with whom?*”, only single classes can be recognized well. For *Mood* and *Problem*, performance is unsatisfactory.

- For *How? Setting* (Figure 4), the classes *Talk* can be recognized with high performance. *With multiple people*, *With one person*, and *Alone* can be recognized well. Similar to *Activity*, *No-match* shows a high F-Score but also a high fpr. *Phone* and *Talking in noise* can be recognized to a small extent.
- For *Talking with whom?* (Figure 5), *No-match* can be recognized excellently while *Friend* can be recognized well. *Self* and *Partner* could be recognized to a small degree while the performance is bad for all remaining classes.
- *Conversation Type* (Figure 6) shows excellent performance for *No-match* and good performance for *Deep conversation* both in terms of ROC and F-Score. Performance is low for *Small* and *Practical talk* while *Personal disclosure* and *Gossip* could not be recognized.
- For *Activity* (Figure 7), good performance was found for classes *Sleep*, *In transit*, and *Exercising* and *Socializing*. *No-match* is recognized with a high F-Score, but the ROC shows that the associated false positive rate (fpr) is also high. *Eat/Drink*, *Housework*, and *Intoxicated* can be recognized to a small degree. *Working* and *Hygiene* could not be recognized.
- *Mood* (Figure 8) was found to be the most challenging target. Only *Mood:Laugh* can be recognized with acceptable performance. *No-match* has a high F-Score, but high fpr; *Sing* can be recognized to a small degree; *Sigh* and *Mad/Argue* cannot be recognized at all; *Cry* is not included in the figure because there were no samples to test on (see Figure 1).
- For *Problem* (Figure 9), only *Friction* can be recognized well. *No-problem* has a high recall, but suffers from a high fpr. *Background noise* could be recognized to a small degree while performance for *Not wearing the EAR* and *Muffled voice* was unsatisfactory. *Different language* and *Ear across the room* could not be recognized.

## 5. Discussion

Applicability of the proposed pipeline could be demonstrated based on the categories *Activity* and “*How? Setting*”. In general, it could also be shown that the method is applicable when sufficient data support is available. Data availability has proven to be a central issue for the proposed method. For all categories, there is a high correlation (Spearman’s ρ = 0.85, *p* < 0.001) between sample number and recognition performance. This was an expected finding because due to uncontrolled recording, the given data contain a lot of noise hampering classification. Multi-task learning and data augmentation—both known for artificially increasing sample size—did not help overcome this issue.

Notable gaps in classification performance exist between the given categories. While a satisfactory performance could be achieved for multiple classes of *Activity* and “*How? Setting*”, mediocre results were found for “*Talking with whom?*” and “Conversation Type”. For *Mood* and *Problem*, performance was unsatisfactory for most classes. Aside data availability, this is caused by structural reasons. For *Activity* and *How*, acoustic clues are broader and present for a longer time. In both cases, information can be gathered from background noise as well as speech as a general target. In comparison, the recognition of a conversation partner (*Whom?*) requires interpretation of voice, pitch, topic, etc., while the acoustic clues for *Mood* are often very short (*Laugh, Sigh*). A related problem is the selectivity for categories and classes, as, for instance, *Problems* appear in many samples, but are only annotated above a certain (subjective) threshold. Other classes such as *Sleep* and *No-match* or *Talking* and *Talking in noise* are also very hard to distinguish. As annotators were instructed to annotate with certainty, acoustic clues might be overlooked.

It could be shown that the proposed method can be used with limited computational resources, and could, therefore, be integrated with the data recording on a mobile device. However, based on the current performance, a fully automatic coding is not yet possible. Since the proposed method allows tuning the results for precision and a number of classes can be recognized well, the method could be employed to support coding.

## 6. Conclusions

In this work, we propose a deep neural network for the new problem of automatic coding of social behavior and environment from uncontrolled audio recordings. The major challenge is the bad quality of audio recordings resulting from the mode of recording which also poses a significant challenge to human annotators. Further, multiple classes can be true for each sample where acoustic clues for a single class might only present less than a single second in recordings of 30 s duration. To overcome those challenges, a pretrained audio classification model encoding features from a broad range of acoustic events was incorporated in the proposed model and an attention mechanism was used to effectively summarize long audio sequences. Based on the results, it was demonstrated that social behavior and environments can be automatically classified from uncontrolled audio recordings when sufficient training data are available. In particular, the categories social *Activity* and *Setting* can be well predicted. Further, it was demonstrated that the classification pipeline can be based on a low-resource deep learning model without a significant loss in performance enabling an application on mobile devices.

However, the results showed that automatic analysis of uncontrolled audio recordings is a difficult task, which is hampered by ambient noise and misplaced recording devices. Moreover, selectivity between certain problems in the annotation is quite low and hampers classification. Therefore, data sparsity for individual classes—and even entire coding categories—remains an issue for the creation of a fully automatic coding pipeline. As a next step, the current pipeline could be used to support annotation and integrated with an active learning approach [50] to increase the number of currently underrepresented classes. Furthermore, temporal relations between different audio samples could be employed, enabling the estimation of most likely sequences of social behavior rather than punctual estimates. Finally, it has to be investigated whether multi-modal data analysis could improve the classification performance. This includes the transcript but also other sensor data, typically available from mobile devices.

## Figures and Tables

**Table 1:** Overview of used pretrained PANN architectures with number of trainable parameters and Precision on AudioSet.

Name	Features	Parameters	AudioSet
Cnn10	logmel-spec	≈5.2 M	0.380
Wavegram-Logmel Cnn14	Wavegram, logmel-spec	≈81.1 M	0.439
MobileNetV2	logmel-spec	≈4.1 M	0.383

**Table 2:** Hyper-parameters with corresponding default values referred to as the base model.

Name	Value
Optimizer	Adagrad
Dropout	0.0
Weight Freezing	fine-tuned
Feature Merging	attention
Attention Heads	8
PANN	MobileNetV2
Augmentation	No
Task	single

**Table 3:** Optimization result summary for different optimization targets on category Activity against the base model. **Ep**: Epoch, **Cor**: Correct Samples, **H**: Hamming Score, **F1**: F-Score, **M**: Macro average, **W**: weighted average.

			Category Scores			Binary F1	
Test	Config	Ep	Cor	Ham	F1	M-⌀	W-⌀
**Base** **Model**	**pretrained**	**17**	**0.644**	**0.657**	**0.660**	**0.334**	**0.635**
	untrained	46	0.600	0.608	0.611	0.334	0.620
Optimizer	Adam	26	0.644	0.653	0.656	**0.350**	**0.637**
Dropouts	0.25	20	0.623	0.635	0.639	0.339	0.629
	0.5	26	0.610	0.624	0.628	**0.356**	**0.636**
Weight Freezing	frozen	11	0.615	0.627	0.631	**0.338**	0.631
	gradual	47	0.588	0.599	0.603	0.326	0.605
Feature Merging	concatenation	47	0.581	0.592	0.596	0.323	0.602
	average	17	0.621	0.632	0.635	**0.347**	0.633
Attention Heads	1	47	0.626	0.634	0.636	0.308	0.612
	4	20	0.611	0.624	0.629	**0.339**	0.624
PANN Model	Cnn10	47	0.622	0.633	0.637	0.334	0.634
	Wave-Cnn14	26	0.632	0.640	0.643	**0.348**	**0.642**
Augmentation	audio	17	**0.649**	**0.664**	**0.668**	**0.340**	**0.650**
	spectrogram	32	0.598	0.611	0.616	**0.366**	0.623
Multi-task	Adagrad	29	0.598	0.611	0.615	0.333	0.618
	Adam	27	0.630	0.640	0.644	**0.366**	**0.645**
Selected	Adam+Aug	13	0.630	0.639	0.642	0.315	0.640

**Table 4:** Performance evaluation for the selected model architecture on the test (development) set for each category (see Figure 1).

	Category Scores			Binary F1	
Test (Epoch 31)	Cor	H	F1	M	W
**How? (Setting)**	0.442 (0.466)	0.596 (0.624)	0.648 (0.677)	0.518 (0.526)	0.637 (0.656)
**Talking with Whom?**	0.722 (0.741)	0.769 (0.784)	0.789 (0.804)	0.241 (0.252)	0.790 (0.790)
**Conversation Type**	0.776 (0.768)	0.826 (0.826)	0.847 (0.849)	0.332 (0.335)	0.885 (0.884)
**Activity**	0.502 (0.539)	0.647 (0.671)	0.697 (0.716)	0.373 (0.364)	0.679 (0.686)
**Mood**	0.896 (0.906)	0.932 (0.934)	0.945 (0.945)	0.274 (0.278)	0.958 (0.954)
**Problems**	0.726 (0.667)	0.834 (0.793)	0.872 (0.836)	0.259 (0.296)	0.897 (0.865)

## Data Availability

Data cannot be made available due to privacy issues.

## Sample Availability

Samples of the compounds are available from the authors.

## Abbreviations

The following abbreviations are used in this manuscript:

CNN	Convolutional Neural Network
AED	Audio Event Detection
SER	Speech Emotion Recognition
ASR	Automatic Speech Recognition
NLP	Natural Language Processing
PANN	Pretrained Audio Neural Network
EAR	Electronically Activated Recorder
ROC	Receiver Operating Characteristics
fpr	false positive rate
